# The Small Colony Variant of *Listeria monocytogenes* Is More Tolerant to Antibiotics and Has Altered Survival in RAW 264.7 Murine Macrophages

**DOI:** 10.3389/fmicb.2016.01056

**Published:** 2016-07-05

**Authors:** Thomas D. Curtis, Lone Gram, Gitte M. Knudsen

**Affiliations:** Gram Lab, Department of Systems Biology, Technical University of Denmark Kongens Lyngby, Denmark

**Keywords:** *Listeria monocytogenes*, small colony variants, antibiotic tolerance, persister cells, cell invasion, oxidative stress

## Abstract

Small Colony Variant (SCV) cells of bacteria are a slow-growing phenotype that result from specific defects in the electron transport chain. They form pinpoint colonies on agar plates and have a variety of phenotypic characteristics, such as altered carbon metabolism, decreased toxin and lytic enzyme production, aminoglycoside resistance, and increased intracellular persistence. They are clinically relevant in *Staphylococcus aureus* and *Pseudomonas aeruginosa*, serving as a reservoir for recurrent or prolonged infections. Here, we found that a SCV mutant in the foodborne pathogen *Listeria monocytogenes* (strain SCV E18), similar to the high persister mutant phenotype, survived significantly better than the wild type when exposed over a 48-h period to concentrations above Minimal Inhibitory Concentration for most tested antibiotics. SCV E18 survived more poorly than the wildtype in unactivated RAW264.7 macrophage cells, presumably because of its reduced listeriolysin O expression, however, it survived better in reactive oxygen species producing, phorbol 12-myristate 13-acetate-activated macrophages. Although SCV E18 was sensitive to oxygen as it entered the stationary phase, it was significantly more tolerant to H_2_O_2_ than the wild type, which may result from a shift in metabolism, however, further investigation is needed to resolve this. SCV E18 is a spontaneous mutant with a point mutation in the *hemA* gene. A wild type copy of *hemA* was complemented on plasmid pSOG30222, which restored the wild type phenotype. The results reported here suggest that the SCV of *L. monocytogenes* could be of clinical importance and highlight a need for adequate clinical screening for this phenotype, as it could affect antibiotic treatment outcomes.

## Introduction

*Listeria monocytogenes* is a Gram-positive, foodborne pathogen that can cause the rare, but often lethal infection listeriosis. This typically affects pregnant women, neonates, the elderly and the immunocompromised, and can cause mortality rates of up to 34% (Farber and Peterkin, 1991). Upon entry into the gastrointestinal tract, *L. monocytogenes* infects host epithelial cells and immediately escapes the phagosome where it is free to replicate within the cytosol. Using actin-mediated mobility it spreads to neighboring cells and eventually enters the bloodstream, causing systemic listeriosis if unchecked by the innate immune system (Portnoy et al., 2002). Macrophages are a key part of the innate immune response and control the initial infection by engulfing and killing the bacteria with of a variety of antimicrobial compounds, such as reactive oxygen and nitrogen species (Shaughnessy and Swanson, 2007).

Treatment options for listeriosis are limited as many antibiotics are only bacteriostatic against *L. monocytogenes*. Ampicillin is the treatment of choice, and is sometimes used in combination with gentamicin, although the necessity of this supplemental treatment has been debated (Temple and Nahata, 2000; Mitjà et al., 2009). For patients who cannot tolerate ampicillin, a combination of trimethoprim and sulfamethoxazole, known as co-trimoxazole (SXT), or macrolides are substituted. (Temple and Nahata, 2000). While resistance in *L. monocytogenes* is uncommon, particularly for the clinically relevant antibiotics (Morvan et al., 2010), we recently demonstrated that this bacterium can form multi-antibiotic tolerant persister cells (Knudsen et al., 2013). However, it is not known if such cells are formed *in vivo* during infection, which could further complicate antibiotic treatment.

Persister cells are a very small subpopulation of bacteria that enter a dormant-like state making them refractory to most antibiotics. Their tolerance to antibiotics is not genetically inherited, and can be operationally defined by a biphasic killing curve when treated with bactericidal antibiotics and no observed increase in the Minimum Inhibitory Concentration (MIC) (Lewis, 2010). Persister cells are thought to exist in all bacteria and have been linked to recurrent infections with a number of diseases (Maisonneuve and Gerdes, 2014). Their formation can be either stochastic and the result of phenotypic switching from normally growing cells to those with reduced growth rates (Balaban et al., 2004), or they can be induced by certain conditions, such as biofilms (Lewis, 2010) or internalization by macrophages (Helaine et al., 2014). One of the primary genetic mechanisms of persister formation identified so far are toxin/antitoxin (TA) modules, which consist of a stable toxin that slows bacterial growth or metabolism and an unstable antitoxin that neutralizes the activity of the toxin under growth conditions. By repeatedly exposing *Escherichia coli* to high concentrations of ampicillin, Fridman et al. (2014) found that bacteria can also tolerate antibiotics by extending the lag-phase by mutations in a variety of pathways, essentially rendering the cells dormant until the transient antibiotic pressure is removed.

Another potential reservoir of persistent and recurrent bacterial infections is the so-called Small Colony Variant (SCV) (Kahl et al., 2016). The SCV is a slow growing phenotypic variant that forms pinpoint colonies when plated on agar, and is the result of either thymidine auxotrophy caused by mutations in the thymidylate synthase gene (Besier et al., 2007; Chatterjee et al., 2008) or an interruption in the electron transport chain (ETC), specifically resulting from an absence of menadione or hemin biosynthesis and metabolism. These variants can either be fixed, which is the result of a mutation in one of the biosynthesis or metabolism genes for the aforementioned compounds, or transient, alternating between the wild type and SCV phenotypes during replication (Edwards, 2012). Additional characteristics include altered carbon metabolism, reduced toxin and lytic enzyme production, and resistance to aminoglycosides (Proctor et al., 2014). Aminoglycosides have also been shown to select for the SCV phenotype, as the reduction in the membrane potential resulting from the disrupted ETC will lower the active transport necessary for aminoglycosides to cross the membrane (Balwit et al., 1994). The combination of trimethoprim/sulfamethoxazole (co-trimoxazole) has been found to select specifically for thymidine auxotrophic SCVs due to the anti-folate action of the drug (Garcia et al., 2013). While SCVs have been primarily studied in *Staphylococcus aureus*, they have been described in a number of bacteria including *Pseudomonas aeruginosa*, *Eacherichia coli*, *Vibrio cholera*, *Salmonella*, and *Lactobacillus acidophilus* (Proctor et al., 2006). Furthermore, they have been linked to a number of recurrent infections including those caused by *S. aureus* (Proctor et al., 1995), *P. aeruginosa* (Haussler et al., 1999) and *E. coli* (Roggenkamp et al., 1998; for a comprehensive review see: Kahl et al., 2016). Although typically less virulent due to their slower growth and reduced expression of virulence factors, such as toxins and lytic enzymes, their ability to adhere, invade and persist within the host cell increases, which is thought to be a crucial factor in the ability of SCVs to cause recurrent or persistent infections (Sendi and Proctor, 2009).

We have previously found that exposure to sub-lethal concentrations of triclosan with subsequent selection on gentamicin could generate stable *L. monocytogenes* SCV cells (Christensen et al., 2011), which all had mutations in one of the heme biosynthesis or metabolism genes and exhibited the same traits observed in SCVs of other species (e.g., colony size, decreased haemolytic activity, aminoglycoside resistance and altered carbon utilization). Our SCV E18 strain also showed reduced growth typical of SCVs from other species, taking roughly four more hours to reach the same maximal cell density as the wild type strain (∼10^9^ CFU/ml), however, we also observed a sensitivity to aerated growth conditions (Kastbjerg et al., 2014), which to our knowledge has not been shown in SCVs from other species.

The phenotypic switching of the SCV and the persister cell are thought to be part of a bacterial bet-hedging strategy, allowing a small percentage of the population to survive and repopulate following stress exposure (Sousa et al., 2012). These two phenotypes share a number of other characteristics including slowed growth, intracellular persistence and a link to chronic infections. Thus, given these similarities, we speculate that a stable *L. monocytogenes* heme deficient SCV would show a similar tolerance toward a broad range of antibiotics that persister cells do. Furthermore, we sought to determine if *L. monocytogenes* SCVs are better at invading and surviving intracellularly, as they have been shown to in *S. aureus* (Sendi and Proctor, 2009). Taken together, these results will help to determine the clinical significance of Small Colony Variants in *L. monocytogenes*.

## Materials and Methods

### Bacterial Strains and Growth Conditions

*Listeria monocytogenes* strain N53-1 and a mutant thereof (E18) were included in the present study. N53-1 represents a food-processing plant persistent molecular subtype and was isolated from a fish smokehouse (Wulff et al., 2006). Strain E18 [denoted as strain (1) 1-1 in Christensen et al. (2011)] is a stable SCV of N53-1, with a Single Nucleotide Polymorphism (SNP) in the glutamyl tRNA reductase (*hemA*) gene (Kastbjerg et al., 2014), generated through an adaptive evolution experiment where N53-1 was exposed to sub-lethal levels of triclosan and subsequently selected on gentamicin (Christensen et al., 2011). Strains were stored at -80°C and grown on Brain Heart Infusion (BHI; Oxoid CM 1135) agar at 37°C for 24 h. Overnight cultures were achieved by inoculating single colonies in 10 ml of BHI broth (Oxoid CM 1135) and incubating at 37°C with shaking at 250 rpm.

### Preparation of Antibacterial Agents

Fresh antibiotic solutions were prepared for each experiment: ampicillin (dissolved in sterile MilliQ water; Sigma–Aldrich A9518), erythromycin (dissolved in 96% ethanol; Sigma–Aldrich E6376), gentamicin (dissolved in sterile MilliQ water; Sigma–Aldrich G3632), norfloxacin (dissolved in sterile MilliQ with 1% glacial acetic acid; Fluka N9890) vancomycin (dissolved in sterile MilliQ; Sigma–Aldrich V2002) and co-trimoxazole, which is comprised of one part trimethoprim (dissolved in sterile MilliQ with 1% glacial acetic acid; Sigma–Aldrich 92131) and five parts sulphamethoxazole (dissolved in acetone; Fluka S7507). H_2_O_2_ (30% in water; Sigma–Aldrich 216763) was diluted to 20 mM in sterile MilliQ water.

### Minimal Inhibitory Concentration (MIC) Assay

Antibiotic MIC values were determined in BHI broth as previously described (Cockerill et al., 2012). In brief, stationary phase cultures of N53-1 and SCV E18 were adjusted to OD_600_ = 0.2 and further diluted 1000-fold, corresponding to a CFU/ml of 10^5^. The adjusted cultures were tested against norfloxacin, ampicillin, gentamicin, and co-trimoxazole (trimethoprim/sulfamethoxazole) using a twofold dilution series of concentrations ranging from 100 to 0.10 μg/ml in 96-well plates (Thermo Scientific 163320) and incubated at 37°C. MIC for N53-1 and SCV E18 was determined by visual inspection after 24 and 48 h of incubation at 37°C, respectively. Two biological replicates were performed.

### Killing Kinetic Assays

To compare the antibiotic and H_2_O_2_ sensitivity of SCV E18 and N53-1, killing kinetics were performed according to Knudsen et al. (2013). Overnight cultures were diluted 10^6^-fold in 10 ml BHI broth and incubated into early stationary phase for 16 h at 37°C with shaking at 250 rpm. Each culture was then adjusted to an optical density at 600 nm (OD_600_) of 0.2 in 2 ml BHI broth and challenged with antibiotic (30X MIC except nitrofurantoin, which was 10X MIC) at 37°C. Bacterial counts were performed at 0, 4, 10, 24, 48, and 72 h. The antibiotics and concentrations used were norfloxacin (100 μg/ml), ampicillin (185 μg/ml), erythromycin (6 μg/ml), vancomycin (47 μg/ml), and co-trimoxazole (95 μg/ml). Cultures treated with H_2_O_2_ were grown and adjusted as described above, then treated with 20 mM and incubated under stagnant conditions at 37°C for 2 h. Three independent biological replicates were performed for each experiment and the limit of detection was 10^2^ CFU/ml.

### Anaerobic Culturing Conditions

To verify that E18 was sensitive to oxygen, it, along with N53-1, were cultured in anaerobic conditions. Oxygen was removed from the media by autoclaving, followed by incubation in a HP0011 anaerobic jar (Oxoid) with an anaerobe gas generation bag (Sigma–Aldrich 68061) for 24 h. Ten microliters of an overnight culture was added to the anaerobic BHI, along with a new anaerobe gas generation bag and incubated on the lab bench for 3 h to allow for an anaerobic atmosphere to be generated before incubation with shaking. The cultures were grown for 72 h at 37°C with shaking at 200 rpm. Two biological replicates were performed.

### *hemA* Complementation

E18 carries several single nucleotide polymorphisms (SNPs) of which one is in the *hemA* gene (Kastbjerg et al., 2014). To verify the role of the SNP in *hemA* in the SCV E18 strain, a *hemA* complementation strain was constructed. The *hemA* gene with a 128 bp upstream region including the native promoter was amplified from genomic DNA of N53-1 with the forward primer 5′-AAACTCGAGTCATCCGTTAACTCCTCG and the reverse primer 5′-AAAGAATTCATAGAAGGAGTTGGAATGGA, which contained terminal XhoI and EcoRI sites, respectively, using Phusion High Fidelity DNA Polymerase (NEB, M0530S). The pSOG30222 plasmid (Hain et al., 2008) and *hemA* amplicon were digested with XhoI and EcoRI, and ligated overnight using a 3:1 insert to vector ratio, then electroporated into 60 μl of electrocompetent DH5α cells using a MicroPulser (BioRad 165-2100). Transformants were selected on Luria–Bertani plates with 100 μg/ml erythromycin and verified by colony PCR and sequencing (GATC Biotech). Plasmids containing the correct pSOG30222::*hemA* construct, along with an unmodified pSOG30222 control plasmid, were transformed into the SCV E18 strain. Transformants were selected on BHI agar with 5 μg/ml erythromycin and confirmed with colony PCR and sequencing. With the exception of the killing kinetic assay, all complementation experiments were carried out in the presence of 5 μg/ml erythromycin. Colony pictures were taken of each strain with an Olympus BX51 microscope at 40× magnification.

### Macrophage Internalization and Survival Assay

A murine macrophage cell line, RAW 264.7 (Sigma–Aldrich 91062702), was used to test bacterial internalization and intracellular survival using a protocol adapted from Bateman and Seed (Bateman and Seed, 2012). RAW 264.7 macrophages were grown in Dulbecco’s Modified Eagle’s Medium – high glucose (Sigma–Aldrich D6546), 10% heat inactivated fetal bovine serum (Sigma–Aldrich F9665) and L-glutamine (Sigma–Aldrich G7513) in 150cc tissue culture flasks, for 10–15 passages prior to use. A cell scraper was used to disjoin the adherent cells from the surface. RAW 264.7 cells were seeded into 24-well plates at a density of 2 × 10^6^ cells/ml in RAW 264.7 culture media and then incubated for a total of 48 h at 37°C with 5% CO_2_. During the 48 h, one group of RAW 264.7 cells was stimulated for 18 h with 1 ng/ml IFNγ, required for macrophage activation, and a second group was stimulated with 100 nM phorbol 12-myristate 13-acetate (PMA), a known inducer of nicotinamide adenine dinucleotide phosphate (NADPH) oxidase activity (Walloschke et al., 2010) for 2 h, while a third control group was left unstimulated. Prior to infection, IFNγ and PMA were removed from the RAW 264.7 containing wells. Overnight cultures of N53-1 and SCV E18 were diluted 10^6^-fold in 10 ml of BHI media and grown at 37°C for 16 h. *L. monocytogenes* cultures were adjusted to a density of 2 × 10^7^ CFU/ml in PBS and added to the confluent RAW 264.7 containing wells, achieving a multiplicity of infection (MOI) of 10. Plates were centrifuged for 5 min at 1,000 rpm to synchronize the infection and incubated at 37°C for 1 h. The plates were then incubated for an additional 2 h in media containing 100 μg/ml gentamicin, which was reduced to 50 μg/ml gentamicin for the remainder of the incubation. In order to verify that gentamicin did not favor the resistant SCV E18 during the 21 h incubation step, a control experiment was performed using the same methods as the unactivated macrophage experiment, with the exception that the media with 50 μg/ml gentamicin was substituted with PBS. To enumerate the surviving *L. monocytogenes* per well, RAW 264.7 cells were washed twice with PBS, followed by lysis with 0.1% Triton-X 100 (Sigma–Aldrich T8787) and then serial dilutions of the intracellular bacteria were plated on BHI agar plates. Internalization was measured following 3 h of incubation, while survival was measured after 24 h.

### Statistical Analysis

CFU/ml values for each biological replicate were log_10_ transformed prior to statistical analysis. For the killing kinetic and O_2_ sensitivity assays, significant differences between the N53-1 and SCV E18 were determined using a paired *t*-test, where significance is equal to *P* < 0.05. Significance between N53-1 and SCV E18 for the RAW 264.7 internalization and survival assays was determined using a paired *t*-test (*P* < 0.05) with the log_10_ transformed CFU/ml values. Significance for each strain between the RAW264.7 treatment groups was calculated using a one-way ANOVA test for the log_10_ transformed CFU/ml values, where *P* < 0.05 determined significance. Significance between the two strains within each RAW264.7 treatment group was calculated using a paired *t*-test (*P* < 0.05). The limit of detection for all CFU/ml values was 10^2^. All data analysis was performed in Excel. Replicates falling below the detection limit were set to 99 for statistical analysis.

## Results

### SCV E18 Shows Increased Tolerance to Antibiotics

The *L. monocytogenes* SCVs, selected on gentamicin, were resistant to other aminoglycosides (Kastbjerg et al., 2014). To determine if the SCV phenotype conveys resistance to other antibiotics, MIC values of selected antibiotics were tested for both N53-1 and SCV E18, which were found to be the same for the two strains (**Table 1**).

**Table 1:** Minimum Inhibitory Concentration (MIC) values for *Listeria monocytogenes* wild type strain N53-1 strain and SCV E18 strain.

	MIC μg/ml					
	
Strain	Ampicillin	Co-trimoxazole	Norfloxacin	Erythromycin	Vancomycin	Gentamicin
N53-1	3.13	0.78	3.13	0.20	1.56	0.40
E18	3.13	0.78	3.13	0.20	1.56	12.50
E18 pSOG30222::*hemA*	1.56	0.78	3.13	–	1.56	0.40
E18 pSOG30222	1.56	0.78	3.13	–	1.56	12.50


*The MIC assay was performed using BHI broth and incubated at 37°C for 24 and 48 h for N53-1 and SCV E18, respectively. All experiments were done with two independent replicates. – , not tested.*

**FIGURE 1:**
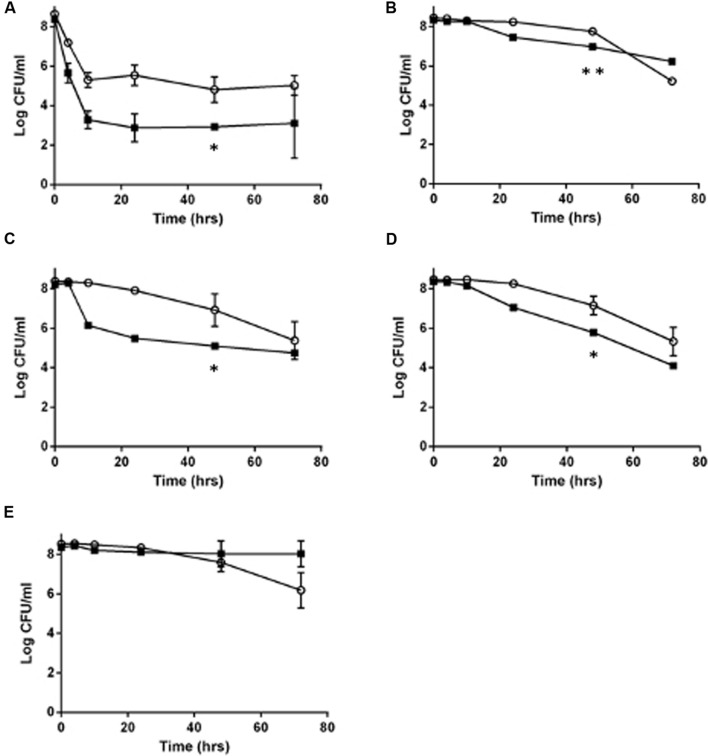
**Killing of *Listeria monocytogenes* N53-1 (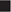) and SCV E18 (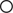) over the course of 72 h under shaking conditions with **(A)** norfloxacin, **(B)** ampicillin, **(C)** co-trimoxazole, **(D)** vancomycin, and **(E)** erythromycin.** Error bars represent standard deviation of the mean for three biological replicates. Significance at the 48-h time point was determined using a paired *t*-test (^∗^*p* < 0.05 and ^∗∗^*p* < 0.005).

Because *L. monocytogenes* SCV grow slower than the wild type (Kastbjerg et al., 2014), and because of the lowered oxidative phosphorylation observed in other SCV organisms, such as *S. aureus* (Proctor et al., 1998), we hypothesized that *L. monocytogenes* SCVs would have an increased tolerance to other classes of antibiotics, which was evaluated using time dependent killing experiments. With the exception of erythromycin, the SCV E18 strain survived significantly better for each antibiotic challenge up to 48 h (**Figure 1**; ampicillin, *P* = 0.0007; co-trimoxazole, *P* = 0.02; vancomycin, *P* = 0.02 and norfloxacin, *P* = 0.03), with between one and threefold higher log_10_ CFU/ml as compared to the N53-1. When treated with norfloxacin, both N53-1 and SCV E18 exhibited a biphasic killing curve with a rapid decline to approximately three and five log_10_ CFU/ml, respectively, which remained stable throughout the 72 h experiment (**Figure 1A**). Ampicillin has a delayed bactericidal effect on *L. monocytogenes* (Winslow et al., 1983), which was also observed with SCV E18, as is evidenced by the roughly three-fold log_10_ reduction in CFU/ml over the 48–72 h time points (**Figure 1B**). However, this delayed bactericidal effect of ampicillin was not observed for N53-1 over the course of the 72 h killing kinetic (**Figure 1B**). By the end of the 72 h treatment with co-trimoxazole, SCV E18 decreased in a linear manner to a comparable level with N53-1 (2.3 × 10^5^ and 5.6 × 10^4^ CFU/ml, respectively; *P* = 0.45; **Figure 1C**). Vancomycin had an initial bacteriostatic effect on both strains, followed by linear killing, however, the bactericidal effect began earlier for N53-1 (*T* = 10), while the SCV E18 strain resisted killing until the 24 h time point (**Figure 1D**). Erythromycin was bacteriostatic on both strains up to the 48 h time point, however, bacterial counts for SCV E18 decreased after 48 h, while the N53-1 remained stable (**Figure 1E**).

While the SCV phenotype in *L. monocytogenes* does not confer notable resistance to antibiotics besides the aminoglycosides, they survived significantly better under high antibiotic concentrations for up to 48 h under ampicillin or co-trimoxazole, and up to 72 h when exposed to norfloxacin or vancomycin. In contrast, by 72 h of exposure to erythromycin or ampicillin, the SCV has fewer CFU/ml than the wild type.

### Oxygen Sensitivity, But H_2_O_2_ Resistance, of the SCV E18

When grown under shaking conditions, SCV E18 grows to a maximum density of approximately 1 × 10^9^ CFU/ml after 12 h, where after the CFU/ml decreases rapidly, eventually dropping below the limit of detection after 24 h (Kastbjerg et al., 2014). Here, we grew cultures of the SCV E18, along with the N53-1, anaerobically to test if the observed decrease in CFU/ml is caused by exposure to oxygen. When grown anaerobically, cultures of the SCV E18 grow to higher densities (8.1 × 10^8^ CFU/ml) after 72 h, than the N53-1 strain (2.5 × 10^8^ CFU/ml; *P* = 0.002) (**Table 2**), suggesting that oxygen is toxic to the SCV E18 strain. Under anaerobic growth conditions the SCV E18 strain retained the pin point colony morphology when plated, whereas the N53-1 strain exhibited variable colony size.

**Table 2:** Oxidative stress sensitivity of *L. monocytogenes* N53-1 and SCV E18 as measured by aerobic and anaerobic growth, and exposure to 20 mM H_2_O_2_ for 2 h.

	Bacterial count (Log_10_ CFU/ml)			
	
	Growth in BHI for 72 h		Survival in 20 mM H_2_O_2_	
		
	Aerobic	Anaerobic	0 h	2 h
N53-1	9.4 ± .01	8.4 ± 0.03	7.2 ± 0.04	3.7 ± 0.09
E18	BD	8.9 ± 0.02	7.2 ± 0.13	6.3 ± 1.02
E18 pSOG30222::*hemA*	8.1 ± 0.09	–	8.0 ± 0.22	5.2 ± 0.17
E18 pSOG30222	BD	–	8.0 ± 0.06	6.5 ± 0.41


*All experiments are log_*10*_ CFU/ml mean values of three biological replicates ± standard deviation. BD, below the limit of detection; –, not tested.*

To test if the oxygen sensitivity was related to a general deficiency in the oxidative stress response, such as the absence of catalase in the SCV E18, we treated with 20 mM H_2_O_2_ for 2 h. Surprisingly, the SCV E18 survived significantly better (*P* = 0.008) than the wild type, with the number of colony forming bacteria following treatment being 33% of the inoculum, whereas the N53-1 strain survived at a rate of 0.03% (**Table 2**). Thus, similar to the increased tolerance toward antibiotics, and regardless of the oxygen sensitivity and lack of catalase (Kastbjerg et al., 2014), the SCV E18 strain exhibits a greater ability to resist killing by H_2_O_2_ over the wild type.

### *hemA* Complementation Restores Wild Type Phenotype of SCV E18

**FIGURE 2:**
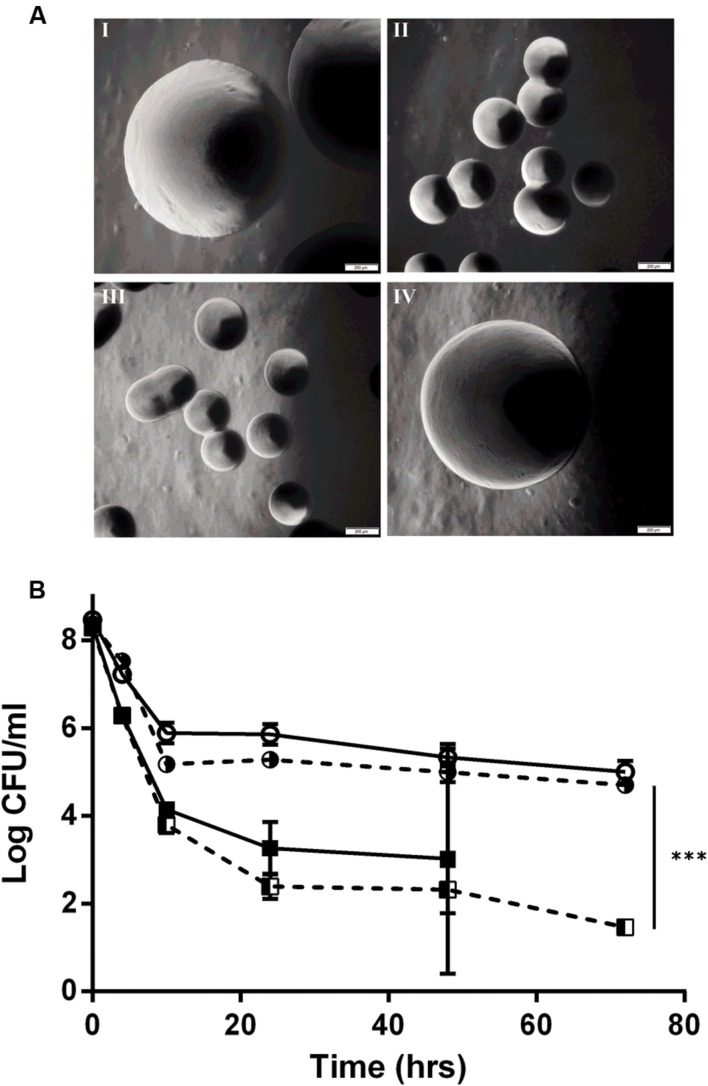
***hemA* complementation experiments showing **(A)** colony morphology at 40x magnification of **I:** N53-1, **II:** SCV E18, **III:** E18 pSOG30222, and **IV:** E18 pSOG30222::*hemA* after 24 h of incubation at 37°C on BHI plates and **(B)** Killing of *L. monocytogenes* N53-1 (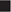) and SCV E18 (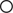) strains, as well as the *hemA* complemented (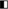) and empty vector control E18 (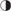) strains exposed to 100 μg/ml norfloxacin for 72 h.** The data represents three biological replicates with standard deviation of the mean. Significance was determined using a paired *t*-test (^∗∗∗^*p* < 0.000005).

By genome sequencing and analyses, we previously demonstrated that all of the *L. monocytogenes* SCVs had several mutations, including a mutation in the *hemA* gene (Kastbjerg et al., 2014). To determine if the tested aspects of the SCV phenotype were due to the SNP in the *hemA* gene, a wild type *hemA* complemented strain of E18 was constructed. This complemented strain regained the large colony phenotype (**Figure 2A**), had restored gentamicin sensitivity identical to the N53-1 strain (**Table 1**), regained sensitivity to H_2_O_2_ and lost its sensitivity to oxygen, as demonstrated by the presence of viable colonies after culturing for 72 h (**Table 2**). Furthermore, the level of antibiotic tolerance was evaluated with a time dependent killing experiment using norfloxacin. The killing curve of the complemented *hemA* strain was significantly different from the empty vector control (*P* = 0.0000046), and was similar to the killing of N53-1 strain, while the empty vector control showed a very similar curve to its non-transformed counterpart SCV E18 (**Figure 2B**).

### SCV E18 Is Less Affected by Macrophage Status Than Wild Type N53-1

**FIGURE 3:**
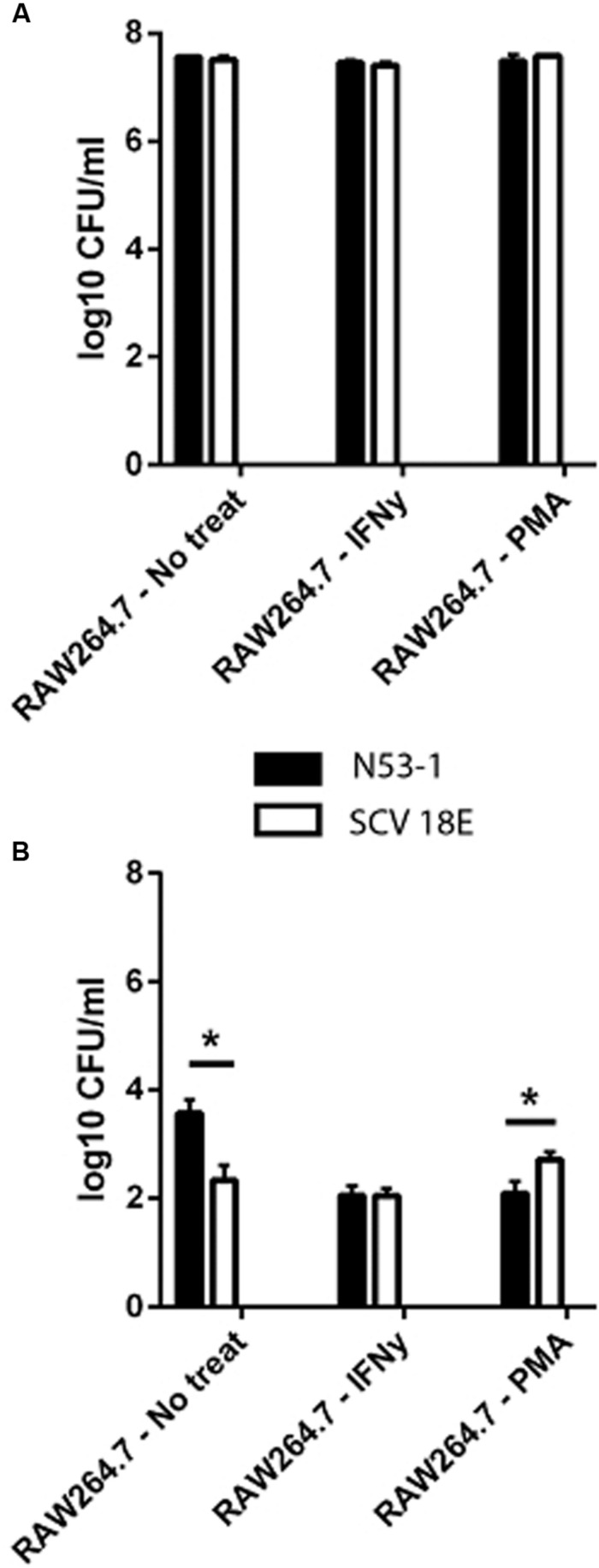
**Intracellular **(A)** internalization and **(B)** survival of *L. monocytogenes* N53-1 and SCV E18 in RAW264.7 cells treated with IFNγ, PMA and no treatment using a multiplicity of infection (MOI) of 10.** Bacterial counts for the internalization assay were determined after 2 h of 100 μg/ml gentamicin exposure. Survival was determined after 21 additional hours of incubation with 50 μg/ml gentamicin. Values are displayed as the log_10_ transformed mean of three biological replicates with standard deviation. Significance was determined using a paired *t*-test (^∗^*p* < 0.05).

We investigated the survival ability of the wild type and the SCV E18 in the IFNγ activated and un-stimulated macrophage model. Additionally, due to the increased tolerance of SCV E18 toward H_2_O_2_, we used macrophages stimulated with PMA, a known activator of the NADPH oxidase pathway. Three hours following infection of the un-stimulated macrophage there was a high degree of internalization and intracellular growth of N53-1 and SCV E18, with 7.6 log_10_ CFU/ml and 7.5 log_10_ CFU/ml recovered, respectively, a difference that was not statistically significant (*P* = 0.77). These internalization findings were similar for the other two treatment groups (**Figure 3A**). By 24 h of incubation in the un-stimulated macrophage, bacterial counts were drastically reduced for both strains, although N53-1 survived significantly better (*P* = 0.006) with 3.6 log_10_ CFU/ml of the N53-1 and 2.3 log_10_ CFU/ml for SCV E18 (**Figure 3B**). When macrophages were activated with IFNγ, the survival difference between strains vanished, as the number of recovered N53-1 bacteria at 24 h was reduced to 2.06 log_10_ CFU/ml, while the SCV E18 burden was almost unchanged by IFNγ activation (2.15 log_10_ CFU/ml) (**Figure 3B**). Increased reactive oxygen species (ROS) production, via PMA activation of the NADPH oxidase pathway, resulted in a slight, but significantly higher bacterial burden of macrophages infected with SCV E18 (2.7 log_10_ CFU/ml), than N53-1 (2.1 log_10_ CFU/ml; *P* = 0.02) (**Figure 3B**). While activation of macrophages with IFNγ or stimulation with PMA significantly reduced the intracellular bacterial load of N53-1 (*P* = 0.01), the SCV E18 was not sensitive to either treatment and remained unchanged across groups (*P* = 0.17).

To summarize, macrophages were equally poor at controlling the bacterial burden of both N53-1 and SCV E18 in the short term (*T* = 3 h), however, by 24 h the CFU/ml for all intracellular bacteria had been severely reduced. While survival in the un-activated macrophage was attenuated for SCV E18, as compared to N53-1, it was unaffected by the status of the macrophage unlike N53-1, which was significantly reduced in CFU/ml upon macrophage activation with IFNγ or stimulation with PMA.

## Discussion

The Small Colony Variant (SCV) has been described for decades in several bacteria such as *S. aureus*, *E. coli* and *P. aeruginosa* (Proctor et al., 2006). However, they were only recently identified in *L. monocytogenes* and, with the exception of aminoglycoside resistance (Kastbjerg et al., 2014), their importance with respect to the treatment of listeriosis has not been investigated. This study is the first to demonstrate that the SCV phenotype of *L. monocytogenes* caused by a single point mutation in the *hemA* gene has the potential to complicate treatment by causing an increase in tolerance toward most of the clinically relevant antibiotics.

As shown previously (Kastbjerg et al., 2014), SCV E18 exhibited resistance to the aminoglycoside gentamicin, a defining trait of the electron-transport deficient SCV (Proctor et al., 2006). This is particularly relevant for listeriosis, as gentamicin is the most common secondary antibiotic used for treatment (Temple and Nahata, 2000). While the SCV E18 showed no increase in MIC for the other antibiotics tested, indicating a lack of acquired resistance, it was nonetheless able to survive lethal concentrations of norfloxacin, ampicillin (up to 48 h), co-trimoxazole, and vancomycin significantly better than the wild type, which taken together, are the definition of antibiotic tolerance (Dimitrijovski et al., 2015). The increase in tolerance toward vancomycin, second generation fluoroquinolones (e.g., norfloxacin) and beta-lactams (e.g., ampicillin) has been observed with *S. aureus* SCVs and is presumably the result of their slow growing SCV phenotype (Chuard et al., 1997; Garcia et al., 2012; Lechner et al., 2012). Antibiotic tolerance poses a unique clinical dilemma, as any testing on isolates relying solely on MIC values could obscure the true antimicrobial susceptibility of the SCV and lead to sub-optimal treatment strategies.

In contrast to the other antibiotics, SCV E18 did not survive better under erythromycin pressure at any time point during the experiment and, as with ampicillin, became more sensitive after 48 h of killing. This could indicate that whatever protective effect the SCV phenotype contributes toward other antibiotics does not apply to the macrolide class, which target protein synthesis. Interestingly, *S. aureus* SCVs were not isolated from cystic fibrosis patients treated exclusively with the macrolide azithromycin, whereas when treated with other antibiotics, SCVs were observed in 46% of patients colonized with *S. aureus* (Kahl et al., 2003; Green et al., 2011). Although the SCV *L. monocytogenes* E18 tolerates ampicillin better than the wild type N53-1 in the short term, the sensitivity toward extended exposure (as evidenced by the pronounced decrease in bacterial count following 48 h of treatment) and the fact that long-term (between 2 and 8 weeks) ampicillin therapy is the primary treatment of choice for listeriosis (Temple and Nahata, 2000), could explain why the SCV phenotype has, to our knowledge, not been observed in in clinical cases. However, it is also possible that current screening procedures in the clinical laboratory are inadequate, leading the SCV sub-population to go unnoticed (Proctor et al., 2006). The slower growth of SCV E18 would likely cause it to be overshadowed in a mixed culture containing the wild type. Furthermore, the altered sugar metabolism and lack of catalase observed in SCV E18 (Kastbjerg et al., 2014), as well as the fact that some of the media used to grow *L. monocytogenes* contain the compounds SCVs are auxotrophic for, would complicate proper identification of any SCV *L. monocytogenes* clinical isolates.

We were expecting the SCV E18 to be more sensitive to H_2_O_2_ because it does not produce catalase, however, it was able to better withstand H_2_O_2_ than the wild type. As H_2_O_2_ reacts with the heme cofactor of proteins (Nagababu and Rifkind, 1998), our observation may simply be the result of fewer targets in the heme deficient SCV E18. Alternatively, tolerance toward H_2_O_2_ may be due to a change in the metabolism of the SCV, as suggested by Painter et al. (2015). In addition to showing that heme auxotrophic SCVs of *S. aureus* were more tolerant of H_2_O_2_, they added 4-hydroxy-2-heptylquinoline-*N*-oxide (HQNO), which can induce the SCV phenotype by blocking the electron transport chain of Gram-positive bacteria, to wild type *S. aureus* either hours or minutes before exposure to lethal levels of H_2_O_2_. Those bacteria grown for hours in the presence of HQNO survived the H_2_O_2_ exposure significantly better than the wild type, whereas those that received the HQNO only minutes before did not. This demonstrates that the tolerance is not simply the result of reduced electron transport, but as the authors suggest, a global physiological change caused by the loss of the electron transport chain (e.g., a switch to anaerobic metabolism), which could also explain our similar observation in *L. monocytogenes*.

In addition to the typical characteristics of the SCV, *L. monocytogenes* SCVs exhibit a unique sensitivity to growth under shaking conditions as they enter the stationary phase (Kastbjerg et al., 2014). Here, we demonstrate that this results from the presence of oxygen in the media. The addition of catalase, which converts H_2_O_2_ to water and oxygen, or heme (necessary for the biosynthesis of catalase) to the growth media abolished this effect, suggesting that the oxygen sensitivity is due to the inability of the heme-deficient SCV mutant to produce catalase (Kastbjerg et al., 2014). However, to our knowledge, this oxygen sensitivity has not been observed in heme-deficient SCVs of other catalase-dependent species (Roggenkamp et al., 1998; Zahra et al., 2013), and this fails to explain why the associated toxicity only manifests in the stationary phase. Few explanations seem to coincide with the observed increased tolerance toward H_2_O_2_ and extended viability of SCV E18 cells when treated with antibiotics, however, we believe this is an important observation that warrants further research.

When the wild type and SCV E18 were inoculated with RAW264.7 murine macrophages over the course of 3 h, we observed no difference between the internalization of the two strains. However, prolonged incubation of the infected macrophages revealed differences between the fates of each strain depending on the activation status of the macrophages. N53-1 survived better than SCV E18 in the naïve macrophages, however, this difference disappeared once macrophages were activated with IFNγ where the wild type was reduced to the same CFU/ml as the mutant. The former observation is likely explained by the reduced expression of the pore-forming toxin Listeriolysin O (LLO) (Kastbjerg et al., 2014) in SCV E18, which has been shown to be produced in murine macrophages (Moors et al., 1999) and is necessary for *L. monocytogenes* to escape from the phagosome (Bielecki et al., 1990). The latter observation corresponds with studies showing that IFNγ-activated macrophages are able to block the escape of *L. monocytogenes* (Portnoy et al., 1989) by a process thought to be mediated by ROS inactivation of the LLO toxin (Myers et al., 2003). This suggests that macrophages need not first be activated by IFNγ in order to control *L. monocytogenes* SCVs, which contrasts to SCVs from other species, and could explain the lack of observed clinical *L. monocytogenes* SCVs. On the other hand, LLO is the most immunogenic antigen for the T cell response, which mediates the ultimate removal and adaptive immunity to *L. monocytogenes* (Vijh and Pamer, 1997), thus a reduction in LLO expression may lead to a weaker adaptive immune response to SCV E18 and could explain the observation that mutants incapable of producing LLO persist for significantly longer in the bone marrow of infected mice (Hardy et al., 2009). Furthermore, the PMA stimulated macrophage showed that SCV E18 is significantly better at surviving the bactericidal effects of ROS within the phagosome, which is consistent with its observed increase in H_2_O_2_ tolerance, and could have implications on the fate of SCV *L. monocytogenes* in neutrophils that rely predominantly on NADPH oxidase for their bactericidal activity (Segal, 2005).

Our complementation experiment showed that this phenotype can arise through a single base-pair substitution in the *hemA* gene, and given the prevalence of verified SCV selection pressures *L. monocytogenes* faces both in the environment and during treatment (e.g., triclosan, gentamicin and co-trimoxazole), the probability of exposure to SCV *L. monocytogenes* seems high. Therefore, it is surprising that no clinical cases of SCV associated listeriosis have been reported. Perhaps the long treatment with ampicillin is enough to eradicate the SCV, or the immune system is better able to control SCV *L. monocytogenes*, as indicated by our findings with non-IFNγ activated RAW264.7 murine macrophages. However, it seems equally likely that this elusive phenotype has gone unnoticed due to a lack of adequate screening procedures in clinical laboratories.

Much like persister cells, SCVs have also been shown to revert to the wildtype phenotype. When clinical and laboratory strains of *S. aureus* were cyclically grown with and without gentamicin, an identical proportion of SCVs upon each exposure to the antibiotic was observed (Massey et al., 2001). Drenkard and Ausubel (2002) found the same phenotypic switching in *P. aeruginosa*, and also identified a protein that modulates the switch between the wild type and SCV phenotypes. This phenotypic heterogeneity, often referred to as a bet-hedging strategy, allows a portion of the bacterial population to survive a stress (e.g., antibiotic exposure or the phagosome), without giving up any fitness advantages associated with mutation. It is plausible that the majority of SCVs *in vivo* are the result of phenotypic switching, and although, we observed no reversion with SCV E18, this mutant and others like it could represent a small subset of SCVs locked in the on position. This would add another treatment challenge, as the bacterial population as a whole would be protected from antibiotics, while simultaneously capable of full virulence (Tuchscherr et al., 2011). Reports of SCVs from a variety of bacterial species being linked to persistent and chronic infections are numerous (Proctor et al., 1995; von Eiff et al., 1997; Roggenkamp et al., 1998; Seifert et al., 1999; Häussler et al., 2003), and while uncommon, cases of recurrent listeriosis have been reported and the cause of these treatment failures were not determined (McLauchlin et al., 1991; Sauders et al., 2001; Kleemann et al., 2009).

Although *L. monocytogenes* SCVs may be unique in that macrophages are able to control them without first becoming activated by IFNγ, they may yet be able to better survive other aspects of the immune system, and the presence of an easily generated, multi-antibiotic tolerant sub-population is nonetheless a cause for concern. Awareness and adequate screening for SCVs in clinical isolates, such as extended incubation times and inclusion of isolates with non-standard sugar metabolism or those lacking catalase activity, would also help to determine the role (if any) this phenotype plays in human infection with *L. monocytogenes*, and we believe the evidence presented here warrants such measures being employed in clinical laboratories.

## Author Contributions

Conceived and designed the experiments: TC, LG, and GK. Performed the experiments: TC. Analyzed the data: TC, LG, and GK. Wrote the manuscript: TC, LG, and GK.

## Conflict of Interest Statement

The authors declare that the research was conducted in the absence of any commercial or financial relationships that could be construed as a potential conflict of interest.
